# A *Caenorhabditis elegans* model of autosomal dominant adult-onset neuronal ceroid lipofuscinosis identifies ethosuximide as a potential therapeutic

**DOI:** 10.1093/hmg/ddac263

**Published:** 2022-10-25

**Authors:** Eleanor Barker, Alan Morgan, Jeff W Barclay

**Affiliations:** Department of Molecular Physiology & Cell Signalling, Institute of Systems, Molecular and Integrative Biology, University of Liverpool, Crown St, Liverpool L69 3BX, UK; Department of Molecular Physiology & Cell Signalling, Institute of Systems, Molecular and Integrative Biology, University of Liverpool, Crown St, Liverpool L69 3BX, UK; Department of Molecular Physiology & Cell Signalling, Institute of Systems, Molecular and Integrative Biology, University of Liverpool, Crown St, Liverpool L69 3BX, UK

## Abstract

Autosomal dominant adult-onset neuronal ceroid lipofuscinosis (ANCL) is a rare neurodegenerative disorder characterized by progressive dementia and premature death. Four ANCL-causing mutations have been identified, all mapping to the *DNAJC5* gene that encodes cysteine string protein α (CSPα). Here, using *Caenorhabditis elegans*, we describe an animal model of ANCL in which disease-causing mutations are introduced into their endogenous chromosomal locus, thereby mirroring the human genetic disorder. This was achieved through CRISPR/Cas9-mediated gene editing of *dnj-14*, the *C. elegans* ortholog of *DNAJC5*. The resultant homozygous ANCL mutant worms exhibited reduced lifespans and severely impaired chemotaxis, similar to isogenic *dnj-14* null mutants. Importantly, these phenotypes were also seen in balanced heterozygotes carrying one wild-type and one ANCL mutant *dnj-14* allele, mimicking the heterozygosity of ANCL patients. We observed a more severe chemotaxis phenotype in heterozygous ANCL mutant worms compared with haploinsufficient worms lacking one copy of CSP, consistent with a dominant-negative mechanism of action. Additionally, we provide evidence of CSP haploinsufficiency in longevity, as heterozygous null mutants exhibited significantly shorter lifespan than wild-type controls. The chemotaxis phenotype of *dnj-14* null mutants was fully rescued by transgenic human CSPα, confirming the translational relevance of the worm model. Finally, a focused compound screen revealed that the anti-epileptic drug ethosuximide could restore chemotaxis in *dnj-14* ANCL mutants to wild-type levels. This suggests that ethosuximide may have therapeutic potential for ANCL and demonstrates the utility of this *C. elegans* model for future larger-scale drug screening.

## Introduction

Neuronal ceroid lipofuscinosis (NCL) comprises a group of progressive diseases characterized by neurodegeneration and lipofuscin accumulation (1,2). NCL usually appears in childhood and can be caused by mutations in a variety of different genes, inheritance of which is almost always recessive. The only autosomal dominant form, adult-onset neuronal ceroid lipofuscinosis (ANCL), also known as CLN4B, Parry disease and autosomal dominant Kufs disease, is unusual in that it is adult-onset and does not affect the visual system, unlike most other NCLs (1,3). ANCL is a rare neurodegenerative disease with a broad clinical variability, commonly presenting with progressive dementia, generalized seizures and movement disorders, which manifest between 20 and 46 years of age, ultimately resulting in death around the age of 45 (3–6). At the cellular level, ANCL is characterized by neurodegeneration, and accumulation of lipofuscin (7). To date, four ANCL-causing mutations have been identified, all of which map to the *DNAJC5* gene, encoding cysteine string protein α (CSPα).

**Figure 1:**
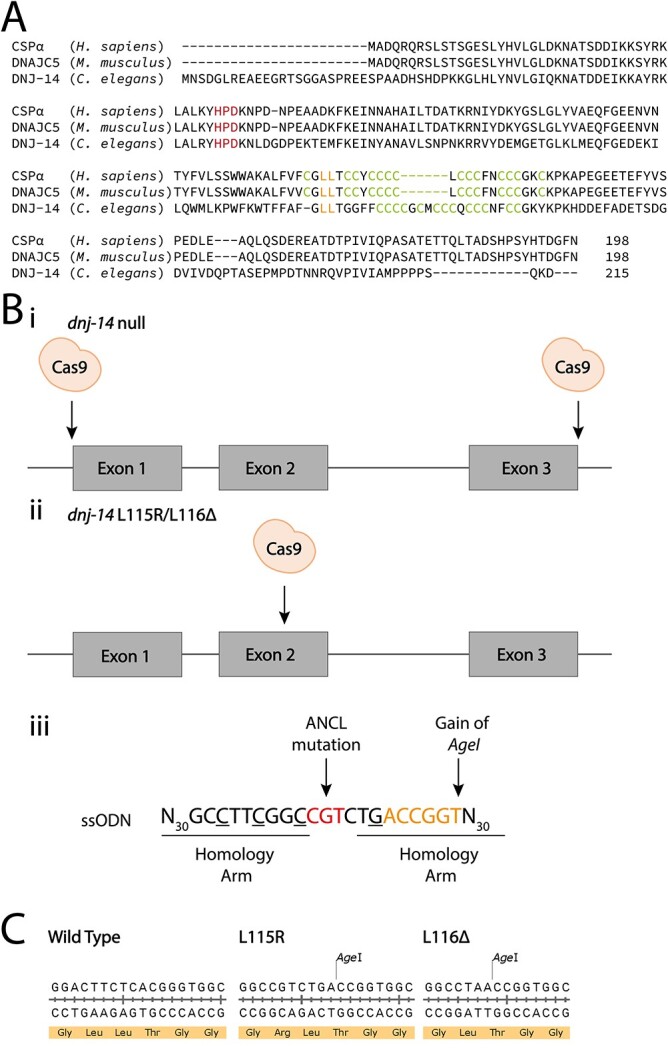
Schematic of DNAJC5 sequence alignment and *dnj-14 C. elegans* mutants CRISPR-Cas9 strategy. (**A**) CLUSTAL multiple sequence alignment of the predicted protein products of the human *DNAJC5* gene (CSPα) with its mouse (DNAJC5) and nematode (DNJ-14) orthologs. The conserved HPD tripeptide motif, dileucine motif and cysteine string are highlighted in red, orange and green, respectively. (**B**) CRISPR-Cas9 design strategy, showing relative position in the *dnj-14* gene of Cas9 cut sites, for *dnj-14(null)* (i) and *dnj-14(L115R)*/*dnj-14(L116Δ)* mutants (ii). (iii) Single-stranded oligonucleotide (ssoligo) repair templates for *dnj-14(L115R)*/*dnj-14(L116Δ)* mutants, with desired ANCL mutation and introduced *AgeI* restriction site shown in red and orange, respectively. Mutations were also introduced to prevent further cutting by Cas9 (underlined) and remove a *BstUI* restriction site. All of these were silent with the exception of a phenylalanine 136 to leucine substitution. This was deemed unlikely to have any effect, as this phenylalanine is very poorly conserved through evolution (it is the only residue not conserved between human and mouse CSP, in which it is a valine; see A), and is an isoleucine in other *Caenorhabditis* species. (**C**) Sequencing results of *dnj-14* mutants indicating the successful introduction of *AgeI* restriction enzyme cut site, and either substitution of leucine to arginine in the *dnj-14(L115R)* mutant or deletion of leucine in *dnj-14(L116Δ)* mutant, compared with that of the wild-type sequence.

DNAJC5/CSPα is a member of the DnaJ/Hsp40 family of co-chaperones that is highly enriched in neurons, where it mainly localizes to synaptic vesicles (8,9). CSP proteins are ubiquitous in metazoans and comprise three conserved domains. The N-terminal J domain contains a characteristic histidine, proline, aspartic acid (HPD) motif, facilitating the binding of CSP to its co-chaperone Hsc70. Connecting the J domain by a conserved linker region is the eponymous cysteine string domain, containing 12–15 cysteine residues (Fig. 1A). Most, if not all, of these cysteines are palmitoylated, and this post-translational modification is essential for the association of CSP to post-Golgi membranes such as synaptic vesicles (10,11). Interestingly, all four ANCL-causing mutations cluster around the cysteine string domain. The two most common variants are point mutations in a dileucine motif immediately preceding the cysteine string: L115R and L116Δ (4,6,12,13) (Fig. 1A). The third is a large 30-base duplication of the cysteine string region: Cys124-Cys133dup (14). Very recently, a fourth mutation also residing within the cysteine-string domain has been identified, which is a missense mutation: C128Y (5).

The major neuroprotective function of CSP is believed to be chaperoning presynaptic proteins, notably by preventing misfolding of the SNARE protein SNAP-25 (15,16). Another distinct neuroprotective role has been proposed for CSP, as a downstream mediator in misfolding-associated protein secretion (MAPS) (17–20). In this process, misfolded proteins, such as α-synuclein and tau, are recruited onto the endoplasmic reticulum (ER) by USP-19 and deubiquitylated, switching their fate from proteasomal degradation to release from the cell via unconventional secretion in a CSP-regulated manner (17–20). More recently, CSP has additionally been implicated in another similar, but functionally distinct mechanism to MAPS, endolysosomal microautophagy (19). These supplementary protein quality control pathways are considered to function in conjunction with the proteasome to reduce the individual cell demand of refolding and/or degrading misfolded proteins, thus preventing the build-up of misfolded/toxic proteins that would result in neuronal dysfunction.

ANCL-causing mutations in CSP are broadly accepted to trigger CSP oligomerization, in addition to causing diffuse cytoplasmic mislocalization (4,14,21–23). Oligomerization was recently reported to occur via ectopic misloading of iron sulphur (Fe-S) clusters by iron–sulphur cluster assembly enzyme onto mutant CSP, as a consequence of reduced palmitoylation in the mutant, and subsequently triggering its mislocalization (22). Notably, this is contradictory to previous findings indicating palmitoylation is required for mutant CSP oligomerization (23,24). The mislocalized oligomerized mutant CSP has been proposed to recruit wild-type CSP into the oligomers in a dominant-negative pathogenic mechanism and thus is no longer able to chaperone SNAP-25 at the synapse, leading to neurodegeneration (22). Interestingly, ANCL-causing mutations are additionally found to interfere with CSP’s role in MAPS, whereas they have no effect on its role in endolysosomal microautophagy (19).

*Caenorhabditis elegans* has been used to model numerous neurodegenerative diseases, because of its facile genetics, extensively characterized nervous system and utility in large-scale drug screening to aid in the identification of potential therapeutic compounds (25). Specifically, *C. elegans* has been utilized previously to study the CSP ortholog, *dnj-14*, which focused on the characterization of two putative null mutant alleles, *dnj-14 (tm3223)* and *dnj-14 (ok237)* (26). Both exhibited reductions in lifespan, defects in neurotransmission and age-dependent impairments of locomotion and neurodegeneration, compared with wild-type controls (26,27). These phenotypes are similar to *csp* null mutant *Drosophila* (8,28) and CSP knockout mice (29), consistent with the widely held view that that CSP performs an evolutionarily conserved universal neuroprotective function(s) (9,30–32). However, the existing *dnj-14* putative null alleles are not ideal. The *ok237* allele deletes the promoter region and the majority of the coding sequence of *dnj-*14, but it extends to remove the N-terminal domain of the adjacent *glit-1* gene, which is required to prevent dopaminergic cell death and protects against oxidative stress (33). Although the *tm3223* allele is confined to *dnj-14*, it is predicted to produce a truncated protein containing the entire N-terminus including the J domain, which may be able to function independently. Furthermore, in addition to the issue that neither of these *dnj-14* alleles provides a specific and complete molecular null, the phenotype of the *dnj-14(tm3223)* mutant has recently been contested (34).

Although CSP null mutants have been studied in flies, mice and *C. elegans,* the only published specific ANCL animal model is a recent study using *Drosophila* (35). This used a knockout-rescue approach, whereby null *csp* alleles were rescued with transgenic fly or human CSP constructs driven by heterologous neuronal-specific promoters. Ideally, however, ANCL-causing mutations should be introduced into the endogenous chromosomal locus, in order to mimic the human genetic disease as closely as possible. Thus, in this study, we used CRISPR/Cas9 gene editing to generate a true *dnj-14* molecular null and two specific ANCL mutants in *C. elegans*. We report the phenotypic characterization of these new mutant strains and demonstrate their potential for therapeutic drug screening, by identifying ethosuximide as being able to rescue the chemotaxis defect in ANCL mutant *C. elegans*.

## Results

### Homozygous *dnj-14* mutants exhibit reduced lifespan and severely impaired chemotaxis

The *C. elegans* genome encodes a single ortholog of the *DNAJC5* gene, *dnj-14*, to which it bears extensive sequence homology (Fig. 1A). Importantly, the two leucines that are mutated in most cases of ANCL reported to date are conserved between worm and human. Given the issues with existing *dnj-14* null mutants, we utilized CRISPR/Cas9 genome editing to create a *dnj-14* null allele with the entire *dnj-14* coding region (exons 1–3 inclusive) removed. This was achieved through sgRNA targeting the 5′ and 3′ ends of the *dnj-14* open reading frame and therefore subsequently directing the Cas9 cut sites (Fig. 1B). No repair template was introduced, thus allowing the DNA to repair itself through non-homologous end-joining. Additionally, the two most common ANCL-causing mutations (L115R and L116Δ in human DNAJC5) were reproduced in their endogenous locus, corresponding to L138R and L139Δ in *C. elegans dnj-14* (hereafter referred to as *dnj-14(L115R)* and *dnj-14(L116Δ)*). This involved utilising sgRNA targeting the Cas9 to cut in exon 2 of *dnj-14*, with a single stranded oligonucleotide (ssoligo) repairing the template with the desired ANCL-causing mutation through homology directed repair (Fig. 1C). Gene edited *C. elegans* lines were identified initially by PCR and restriction fragment analysis (Supplementary Material, Fig. S1), followed by DNA sequencing to confirm that the intended edits had been successfully introduced.

**Figure 2:**
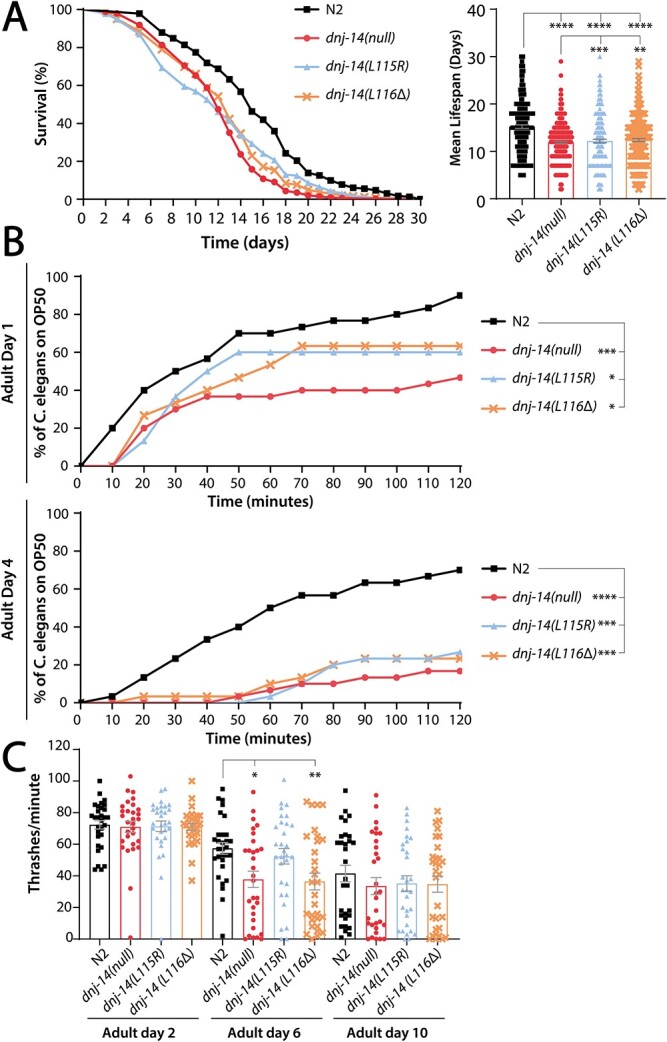
Homozygous *dnj-14* mutants have shortened lifespans, impaired chemotaxis and age-dependent defects in locomotion. (**A**) *dnj-14* homozygous mutants have a shortened lifespan at 20°C, compared with Bristol N2 controls. Data were pooled from three independent biological replicates and are shown as survival curves (left panel) and bar charts of mean lifespan ± SEM with raw datapoints overlaid (right panel). Mean lifespans in days of adulthood were as follows: N2 = 17.16 (*n* = 231), *dnj-14(null)* = 12.95 (*n* = 342), *dnj-14(L115R)* = 14.61 (*n* = 232), *dnj-14(L116Δ)* = 13.99 (*n* = 262). Significant differences were found between wild-type N2 and all three *dnj-14* mutants; between *dnj-14(null)* and *dnj-14(L115R)*, and between *dnj-14(null)* and *dnj-14(L116Δ)*. There was no significant difference in lifespan between the *dnj-14(L115R)* and *dnj-14(L116Δ)* ANCL mutants. (**B**) *dnj-14* homozygous mutants exhibit impaired chemotaxis at day 1, which progresses by day 4 of adulthood. Chemotaxis was assayed through measuring the time taken for worms to reach a bacterial food source 30 mm away. A minimum of 30 worms were assayed per strain, from three independent biological repeats. Significant differences were found between wild-type N2 and all three *dnj-14* mutants at both day 1 and 4. There were no significant differences between any of the three *dnj-14* mutants. (**C**) *dnj-14(null)* and *dnj-14(L116Δ)* exhibit age-dependent defects in locomotion. Locomotion was determined by thrashing in solution per minute, assaying at day 2, 6 and 10 of adulthood. *dnj-14(null)* and *dnj-14(L116Δ)* exhibited significant defects in locomotion at adult day 6 in comparison with N2 controls, but there were no other significant differences between strains. Data are shown as mean ± SEM, with 30 worms assayed per strain per timepoint from three independent biological repeats. For all panels, *P*-values for statistically significant differences between strains are indicated as follows: ^*^^*^^*^^*^P < 0.0001, ^*^^*^^*^P < 0.001, ^*^^*^P < 0.01, ^*^P < 0.05.

As mutations in CSP result in reduced lifespan in humans (4,5,12–14), mice (29), flies (8) and previously characterized *C. elegans dnj-14* mutants (26), we performed a lifespan analysis on our newly created homozygous *dnj-14* mutant and wild-type Bristol N2 worms. In each of three independent experiments, *dnj-14(null)*, *dnj-14(L115R)* and *dnj-14(L116Δ) C. elegans* consistently exhibited significantly shorter lifespans compared with N2 controls (Fig. 2A). The mean lifespan was 12.95 days (95% CI: 12.54–13.36), 14.61 days (95% CI: 13.92–15.30) and 13.99 days (95% CI:13.42–14.55) for the *dnj-14(null)*, *dnj-14(L115R)* and *dnj-14(L116Δ)* strains, respectively, compared with 17.16 days (95% CI: 16.50–17.83) for the wild-type N2 control strain. Thus, either the loss of *dnj-14* or ANCL-causing mutations in *dnj-14* results in a reproducible lifespan reduction. However, the *dnj-14(L115R)* and *dnj-14(L116Δ)* strains were significantly longer lived than the null mutant (*P* < 0.0001 and *P* < 0.0061, respectively), suggesting that the ANCL mutations are not complete loss-of-function alleles (35).

**Table 1:** Summary of phenotypic characterization of homozygous *dnj-14* mutants

Phenotype tested	Assay	Phenotype observed		
		***dnj-14(null)***	***dnj-14(L115R)***	***dnj-14(L116Δ)***
Lifespan	**Lifespan**	**25% reduction in mean lifespan (*P* < 0.0001).**	**15% reduction in mean lifespan (*P* < 0.0001)**	**18% reduction in mean lifespan (*P* < 0.0001)**
Chemosensation	**Timed food Race**	**76% reduction in worms reaching bacterial food source after 2 h, at adult day 4 (*P* < 0.0001)**	**62% reduction in worms reaching bacterial food source after 2 h, at adult day 4 (*P* < 0.0001)**	**67% reduction in worms reaching bacterial food source after 2 h, at adult day 4 (*P* < 0.0001)**
Locomotion	**Thrashing assay**	**34% reduction in locomotion at adult day 6 (*P* < 0.05)**	No locomotion defect observed by day 10 of adulthood	**37% reduction in locomotion at adult day 6 (*P* < 0.01)**
Effect of ethanol on locomotion	**Ethanol thrashing assay**	**Loss of stimulatory response of 17 mM ethanol on locomotion at adult day 2 (*P* < 0.001)**	**Loss of stimulatory response of 17 mM ethanol on locomotion at adult day 2 (*P* < 0.01)**	**Loss of stimulatory response of 17 mM ethanol on locomotion at adult day 2 (*P* < 0.01)**
Seizure-like activity	**PTZ assay**	No PTZ-induced seizure-like activity observed by day 10 of adulthood	No PTZ-induced seizure-like activity observed by day 10 of adulthood	No PTZ-induced seizure-like activity observed by day 10 of adulthood
Mechanosensation	**Touch sensitivity assay (anterior touch response)**	No change in mechanosensation by day 10 of adulthood	No change in mechanosensation by day 10 of adulthood	No change in mechanosensation by day 10 of adulthood
Pharyngeal pumping	**Electropharyngeograms (EPGs)**	No change mean EPG, or inter-pump-interval at day 1 or 5 of adulthood	No change mean EPG, or inter-pump-interval at day 1 or 5 of adulthood	No change mean EPG, or inter-pump-interval at day 1 or 5 of adulthood
Brood size	**Self-brood size assay**	No change in overall self-brood size		

Previous studies of the *tm3223* and *ok237* alleles reported a progressive, ageing-dependent impairment of chemotaxis (26,27). We therefore utilized the same assay, in which the time taken for worms to move to an attractive bacterial food source is measured. All three of our new homozygous *dnj-14* mutant worm strains exhibited significant impairments in chemotaxis (Fig. 2B). This defect was evident from the start of adulthood at adult day 1 and deteriorated progressively to adult day 4, by which time there was a severe 62–76% reduction in worms detecting and reaching the food source after 2 h. Next, we set out to investigate a different phenotype that is mediated by chemosensory neurons: the stimulatory effect of low concentrations of ethanol on locomotion, which acts via IL2 chemosensory neurons (36). We found that, in contrast to wild-type N2 controls, all three homozygous *dnj-14* mutant *C. elegans* were unaffected by ethanol (Supplementary Material, Fig. S2A). This was evident at both adult day 2 and 5, at which point there was an absence of the stimulatory effect of 17 mM ethanol on thrashing. Mutation of *dnj-14* did not appear to affect the function of all neurons, however, as mechanosensory neuron activity (assayed through anterior touch response) was unaffected at day 2, 6 or 10 of adulthood (Supplementary Material, Fig. S2B). Similarly, the activity of pharyngeal neurons (measured through electropharyngeograms (EPGs)) revealed no change at either day 2 or 5 of adulthood between the *dnj-14(null)* worms and N2 wild-type controls, whether utilising either mean EPG frequency or inter-pump-interval time as electrophysiological readouts of pharyngeal activity (Supplementary Material, Fig. S2C–D).

Another commonly reported symptom of ANCL is ataxia (4); therefore, we assayed for defects in locomotion. Although *dnj-14* mutants exhibited superficially normal movement on agar plates, when assaying locomotion through thrashing in solution, we observed a small age-dependent reduction in locomotion (Fig. 2C). This defect was not present when observing young adult day 1 worms, however, and only manifested when the worms were aged to day 6 of adulthood. Notably, this small reduction in locomotion was not present in the *dnj-14(L115R)* mutant strain. Next, we reasoned that as seizures are another frequently observed symptom of ANCL, we could use an established assay for sensitivity to pentylenetetrazol (PTZ)-induced seizure-like activity (37–39). However, we did not observe PTZ-induced convulsions in any of the *dnj-14* mutant strains; whereas the GABA _A_ receptor *unc-49(e407)* mutant, used as a positive control, exhibited a high susceptibility to PTZ-induced head-bobbing convulsions in the same experiments (Supplementary Material, Fig. S3A).

Finally, fecundity was investigated, as this was previously reported to be greatly reduced in *dnj-14 (ok237)* mutant worms (27), assayed through measuring the total number of offspring produced as a result of selfing. However, we did not identify a difference in the overall brood size between the homozygous *dnj-14(null)* worms and wild-type N2 controls (Supplementary Material, Fig. S3B). Thus, we concluded that defects in the brood size were unlikely to be present in the *dnj-14(L115R)* and *dnj-14(L116Δ)* ANCL mutants, if absent from the *dnj-14(null)*. Despite not identifying any difference in the overall brood size, we observed a small temporal delay in egg laying in the *dnj-14(null)*, compared with the wild-type N2 controls. Three days post-egg lay, wild-type N2 control *C. elegans* produced a significantly larger brood size, compared with *dnj-14(null)* worms. However, this effect was reversed by 5 days post-egg lay (Supplementary Material, Fig. S3B).

### Heterozygous *dnj-14* mutants exhibit reduced lifespan and impaired chemotaxis

Having generated a full phenotypic characterization of the homozygous *dnj-14* mutants, summarized in Table 1, we next sought to investigate the phenotypic profile of heterozygous *dnj-14* mutants in order to establish an accurate disease model, as ANCL is an autosomal dominant condition. This was achieved by crossing homozygous *dnj-14* mutants onto an aneuploidy-free X-chromosome balancer strain covering the *dnj-14* locus (40). Balanced heterozygotes harbouring both wild-type and mutant *dnj-14* alleles were distinguished from homozygotes via the recessive *lon-2* marker and the dominant *Pmyo-2*::*mCherry* marker in the balancer, by selecting worms with pharyngeal mCherry fluorescence that lacked the *Lon* phenotype. We also crossed our N2 cultivar with this balancer strain to produce an accurate wild-type comparator strain, to control for any effects of the various structural rearrangements and transgenes contained within the balancer chromosome. Heterozygosity for *dnj-14* mutations in the balanced strains was confirmed at the molecular level by PCR and restriction fragment analysis prior to phenotypic analysis (Supplementary Material, Fig. S1).

**Figure 3:**
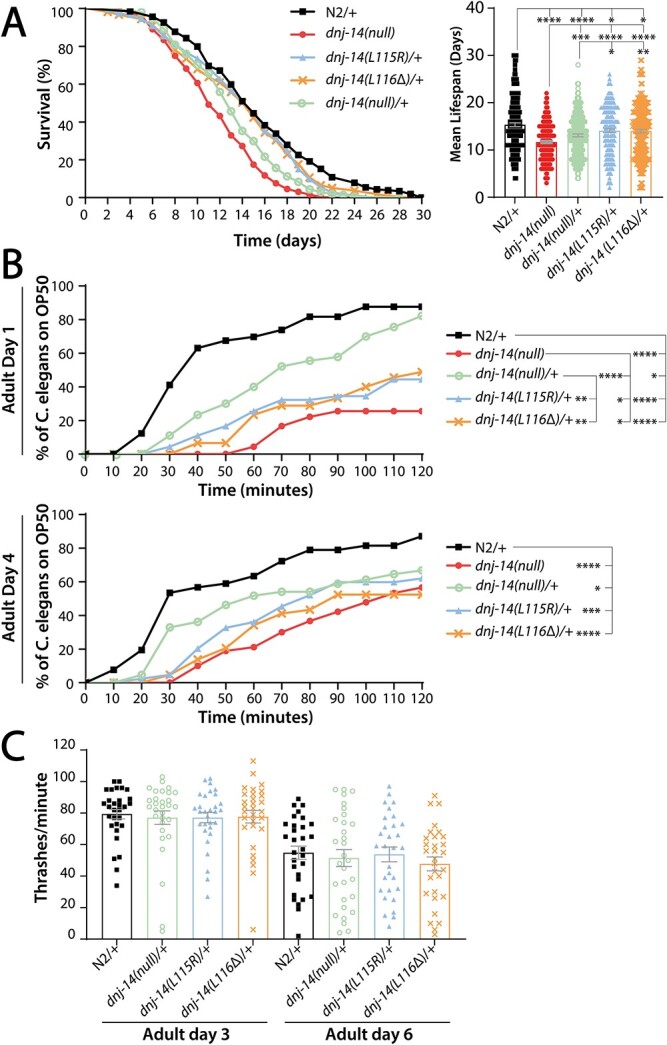
Heterozygous *dnj-14* mutants have reduced lifespans and impaired chemotaxis. (**A**) *dnj-14* heterozygous mutants have a shortened lifespan at 20°C, compared with Bristol N2 controls. Data were pooled from three independent biological replicates and are shown as survival curves (left panel) and bar charts of mean lifespan ± SEM with raw datapoints overlaid (right panel). Mean lifespan in days of adulthood were as follows: N2/+ = 15.8 (n = 203), *dnj-14(null)* = 11.73 ± (*n* = 264), *dnj-14(null)/+* = 13.3 (*n* = 204), *dnj-14(L115R)/+* = 14.49 (*n* = 185), *dnj-14(L116Δ)/+* = 14.38 (*n* = 215). Significant differences were found between wild-type N2/+ balanced heterozygotes and all four *dnj-14* homozygous and heterozygous mutants; between homozygous *dnj-14(null)* and all three heterozygous *dnj-14* mutants; and between heterozygous *dnj-14(null)/+* and heterozygous *dnj-14(L115R)/+* mutants and *dnj-14(L116Δ)/+* mutants. There was no significant difference in lifespan between heterozygous *dnj-14(null)/+* and *dnj-14(L116Δ)/+* mutants, or between the *dnj-14(L115R)/+* and *dnj-14(L116Δ)/+* ANCL mutants. (**B**) *dnj-14(L115R)/+* and *dnj-14(L116Δ)/+* worms have significantly impaired chemotaxis ^*^^*^^*^^*^(*P* < 0.0001). Chemotaxis defects were assayed through measuring the time taken for worms to reach a bacterial food source a defined distance away. A minimum of 32 worms were assayed per strain, from three independent biological replicates. At day 1, significant differences in chemotaxis were found between wild-type N2/+ balanced heterozygotes and all four *dnj-14* homozygous and heterozygous mutants; between homozygous *dnj-14(null)* and all three heterozygous *dnj-14* mutants; between heterozygous *dnj-14(null)/+* and both heterozygous *dnj-14(L115R)/+* and *dnj-14(L116Δ)/+* mutants, but there was no significant difference in chemotaxis between the *dnj-14(L115R)/+* and *dnj-14(L116Δ)/+* ANCL mutants. At day 4, significant differences in chemotaxis were found between wild-type N2/+ balanced heterozygotes and all four *dnj-14* homozygous, but not between any other strains. (**C**) Heterozygous *dnj-14* mutants have no significant defects in locomotion. Locomotion was determined by thrashing in solution per minute, assaying at day 3 and 6 of adulthood. Thirty worms per strain were assayed for each timepoint tested, from three independent biological replicates. For all panels, *P*-values for statistically significant differences between strains are indicated as follows: ^*^^*^^*^^*^P < 0.0001, ^*^^*^^*^P < 0.001, ^*^^*^P < 0.01, ^*^P < 0.05.

We then investigated whether the phenotypes present in the homozygous *dnj-14* mutants were also present in the heterozygous *dnj-14* mutants, namely a reduced lifespan, impaired chemotaxis and a small age-dependent defect in locomotion. We identified a small, but statistically significant reduction in lifespan of *dnj-14(L115R)/+* and *dnj-14(L116Δ)/+*, compared with wild-type N2*/+* worms (Fig. 3A). Interestingly, we identified a greater reduction in the lifespan of *dnj-14(null)/+* worms, although the lifespan reduction was not as severe as that of homozygous *dnj-14(null)* worms. The mean lifespans for the ANCL mutant strains were 15.80 days (95% CI: 15.03–16.57) for the N2*/+* wild-type controls, 14.49 days (95% CI: 13.79–15.18) for *dnj-14(L115R)/+*, 14.38 days (95% CI: 13.64–15.11) for *dnj-14(L116Δ)/+*, 13.30 days (95% CI: 12.72–13.89) for *dnj-14(null)/+* and 11.73 days (95% CI: 11.25–12.20) for the homozygous *dnj-14(null).* Additionally, we identified that the defect in chemotaxis in the homozygous *dnj-14* mutants was also present in the heterozygous *dnj-14* ANCL point mutants (Fig. 3B). In contrast to the findings from the lifespan analysis, we were only able to identify a small chemotaxis defect in the *dnj-14(null)/+* worms. In fact, there was a much greater chemotaxis defect in the heterozygous *dnj-14* ANCL point mutants compared with *dnj-14(null)/+* worms, consistent with a dominant-negative action. However, the small reduction in locomotion identified in two of the homozygous *dnj-14* mutants could not be replicated in the in any of the heterozygous *dnj-14* strains (Fig. 3C).

### Human CSP expression rescues *dnj-14* null, but not ANCL, mutants

Although worm DNJ-14 and human DNAJC5/CSPα share considerable amino acid sequence similarity, they have not been demonstrated to be functional orthologues. We therefore sought to determine whether the defects in the *dnj-14* mutant worms could be rescued through expression of human DNAJC5/CSPα (hCSP). This was achieved by creating a synthetic DNA construct of the human *DNAJC5* coding sequence (codon-optimized for expression in *C. elegans*) and inserting this downstream of the putative *dnj-14* promoter in a previously described vector (26). This was then injected into N2 animals, along with a *Prab-3*::GFP reporter, to enable the selection of worms expressing hCSP in extrachromosomal arrays.

**Figure 4:**
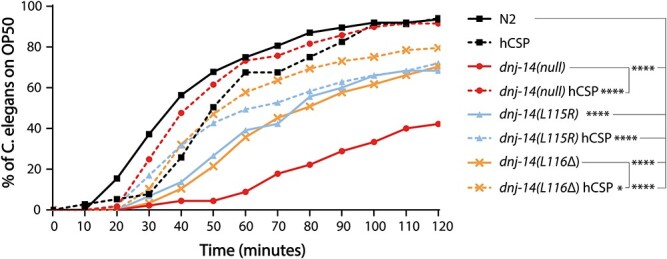
Expression of human CSP fully rescues the *dnj-14(null)* chemotaxis defect. Chemotaxis defects were assayed through measuring the time taken for worms to reach a bacterial food source 30 mm away. Homozygous *dnj-14(null)*, *dnj-14(L115R)* and *dnj-14(L116Δ) C. elegans* exhibited a significant defect in chemotaxis compared with wild-type N2 controls. In contrast, *dnj-14(null)* worms expressing human CSP (hCSP) were not significantly different from wild-type N2 worms, indicating the complete rescue of this phenotype. However, *dnj-14(L115R)* and *dnj-14(L116Δ)* worms expressing hCSP still retained significant defects in chemotaxis compared with wild-type N2 worms, indicating that full rescue was not achieved. Expression of hCSP resulted in a small, but significant, enhancement in chemotaxis in the *dnj-14(L116Δ)* mutant, indicating only a partial rescue of this phenotype. N2 wild-type animals expressing hCSP were utilized as an additional control and were not significantly different from non-transformed N2 worms. Data shown were pooled from assays using a minimum of 32 worms per strain, from three independent biological replicates for the N2, N2 + hCSP, *dnj-14(null) and dnj-14(null)* + hCSP strains; and a minimum of 55 worms per strain, from six independent biological replicates for the *dnj-14(L115R)*, *dnj-14(L115R)* + hCSP, *dnj-14(L116Δ)*, and *dnj-14(L116Δ)* + hCSP strains. *P*-values for statistically significant differences between strains are indicated as follows: ^*^^*^^*^^*^P < 0.0001, ^*^^*^^*^P < 0.001, ^*^^*^P < 0.01, ^*^P < 0.05.

These were then crossed with the homozygous *dnj-14* mutant strains, selecting worms that were GFP positive and homozygous for the appropriate *dnj-14* allele. It is well established that extrachromosomal arrays typically harbour multiple transgene copies (41); hence, the human CSPα protein would be expected to be expressed to at least the same level as endogenous worm DNJ-14. We utilized the chemotaxis assay to determine whether rescue had been achieved. Strikingly, the expression of hCSP was able to fully rescue the chemotaxis defect of homozygous *dnj-14(null)* worms to the levels of the wild-type N2 controls (Fig. 4). This demonstrated that worm and human CSP are functionally interchangeable. Conversely, this was not the case for either the *dnj-14(L115R)* or *dnj-14(L116Δ)* homozygous ANCL point mutants, where no significant improvement and only a partial rescue, respectively, was observed. The inability of human CSP to fully rescue chemotaxis in the ANCL mutants is consistent with the dominant-negative effect we observed in this assay with heterozygous worms (Fig. 3B), suggesting that mutant worm DNJ-14 can interact with wild-type human CSPα to impair its function.

### Screen for compounds that rescue ANCL *dnj-14* mutants identifies ethosuximide

**Figure 5:**
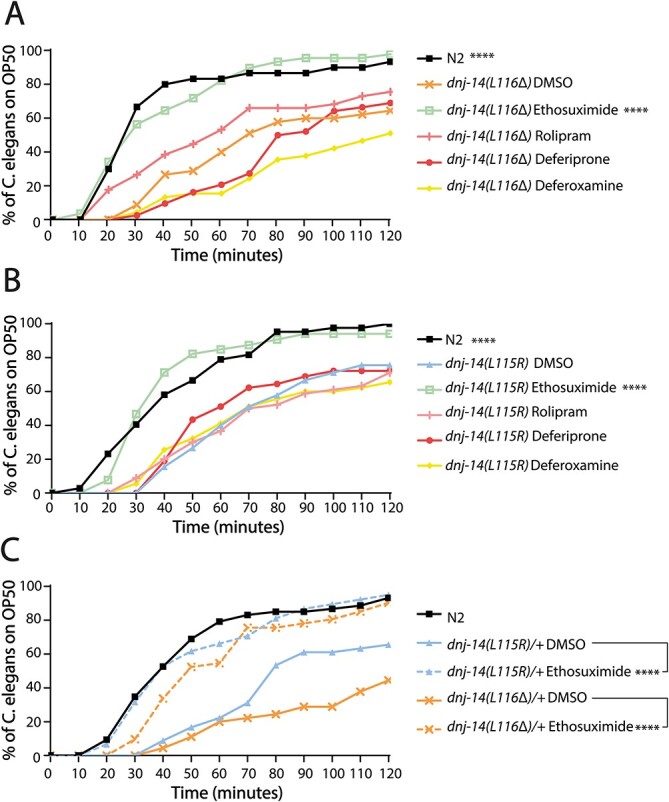
Ethosuximide ameliorates the chemotaxis defect of *dnj-14* ANCL mutants. (**A**–**B**) Drug screen for compounds able to improve the chemotaxis defect of *dnj-14(L115R)* (A) and *dnj-14(L116Δ)* (B) mutants. Worms were grown on NGM plates containing the indicated compounds, or 0.1% DMSO vehicle controls from the point of egg-laying. *dnj-14(L115R)* and *dnj-14(L116Δ)* worms have a significant improvement in chemotaxis response when grown in the presence of ethosuximide (^*^^*^^*^^*^P > 0.0001). (**C**) Chemotaxis assay on *dnj-14(L115R)/+* and *dnj-14(L116Δ)/+* grown either in the presence of 1 mg/mL ethosuximide, or 0.1% DMSO control. Both strains exhibit a significant improvement in chemotaxis when raised in the presence of ethosuximide, compared with that of the DMSO control (^*^^*^^*^^*^P < 0.0001). Chemotaxis was assayed through the time taken for the worms to detect and move to a bacterial food source. A minimum of 30 worms were assayed per strain, per condition, from three independent biological repeats. *P*-values for statistically significant differences between strains are indicated as follows: ^*^^*^^*^^*^P < 0.0001, ^*^^*^^*^P < 0.001, ^*^^*^P < 0.01, ^*^P < 0.05.

Having characterized our worm model of ANCL, we set out to test whether it could be utilized as a tool to identify compounds with therapeutic potential. As a proof of principle, we screened a small number of compounds for their ability to rescue the chemotaxis defect of homozygous *dnj-14* mutants, compared with that of wild-type N2 control worms. Ethosuximide and rolipram were selected as they have previously been reported to rescue the lifespan and chemotaxis phenotype of other *dnj-14* mutant alleles but had no significant effect on wild-type worms (26,27). Additionally, the iron chelating drugs, deferiprone and deferoxamine were selected because of their reported ability to induce a partial rescue of ANCL pathology in cultured cells derived from ANCL patients (22). The initial screen, carried out on *dnj-14(L116Δ)* ANCL mutants, revealed ethosuximide as the only compound able to rescue the chemotaxis defect to the levels of the wild-type N2 controls (Fig. 5A). Consistently, ethosuximide also produced a full chemotaxis rescue when extending the small-scale screening to the homozygous *dnj-14(L115R)* mutant (Fig. 5B).

Having established that ethosuximide can rescue both *dnj-14(L115R)* and *dnj-14(L116Δ)* ANCL homozygous mutant worms, we sought to investigate whether these findings could be extended to that of heterozygous *dnj-14(L115R)/+* and *dnj-14(L116Δ)/+* worms. Similarly, we found that both heterozygous ANCL mutant worms had significant improvement in chemotaxis when grown under the presence of ethosuximide, compared with that of the vehicle control, DMSO (Fig. 5C).

## Discussion

In this study, we report that homozygous *dnj-14(null)* and ANCL mutant *dnj-14(L115R)* and *dnj-14(L116Δ)* worms exhibit a shortened lifespan, impaired chemotaxis and age-dependent defects in locomotion. Notably, several assays performed in this study yielded phenotypes indistinguishable from wild-type N2 controls, thus indicating that these worms are not generally sick. Instead, these models exhibited a specific pattern of age-related phenotypes, similar to human ANCL patients (4–6,13,14). Interestingly, the phenotype of the homozygous *dnj-14(null)* worms was more severe than either of the ANCL mutant *dnj-14(L115R)* and *dnj-14(L116Δ)* worms, both in terms of lifespan and chemotaxis defects. This indicates that ANCL-causing mutations in CSP do not cause a complete loss of function, consistent with findings from a *Drosophila* ANCL model (35), but contrasting with conclusions made from a neuronal cell culture model (22). These reported phenotypes are also comparable with those from CSP knockout mice, which exhibit a shortened lifespan and progressive sensorimotor defects (13). Additionally, the findings from this study mirror those from previously characterized *dnj-14(ok237)* and *dnj-14(tm3223)* alleles (26,27). However, we were unable to reproduce the defects previously reported in brood size in the *dnj-14(ok237)* allele (27). We therefore conclude that this phenotype is likely a consequence of partial deletion of the adjacent *glit-1* gene, or interference with both genes, rather than a direct result of loss of *dnj-14*. This highlights the importance of generating ‘true’ *dnj-14* null alleles to study CSP function, as carried out in this study. Our findings reported here directly oppose those from one study carried out on the *dnj-14(tm3223)* putative null allele, where no effects on the lifespan or locomotion were found (34). The reasons for this discrepancy over the phenotypes of the *dnj-14(tm3223)* allele are unclear. However, as all three of the new mutant alleles created in this study, as well as the *dnj-14(ok237)* and *dnj-14(tm3223)* alleles, have been shown to exhibit significantly reduced lifespan and severely impaired chemotaxis, this strongly suggests that these represent robust and reproducible phenotypes caused by *dnj-14* mutation.

Both phenotypes of a shortened lifespan and defective chemotaxis persisted when outcrossing the *dnj-14* homozygotes to produce heterozygous *dnj-14* mutants. Interestingly, we observed a significantly greater chemotaxis defect in *dnj-14(L115R)/+* and *dnj-14(L116Δ)/+* worms compared with *dnj-14(null)/+* worms, suggesting that one ANCL-mutated copy of *dnj-14* is more harmful than the loss of one copy of *dnj-14*. This evidence corroborates previous suggestions that ANCL-causing mutations have a gain of function, or dominant-negative disease mechanism (4,21–23,35,42,43). This is further supported by our findings that expression of human CSP (hCSP) induces a full chemotaxis rescue of *dnj-14(null)* worms to wild-type levels, but is unable to fully rescue *dnj-14(L115R)* and *dnj-14(L116Δ)* worms. Furthermore, the complete chemotaxis rescue of *dnj-14(null)* worms validates that the chemotaxis defects identified in this study are a direct consequence of the loss of *dnj-14*, and therefore cannot be attributed to off-target effects of CRISPR/Cas9 gene editing.

The chemotaxis assay used here requires worms to both sense a chemical attractant and move to its source. Hence, it is important to ensure that any effects are because of defective chemosensation, rather than impaired motor function. Reassuringly, the observed chemotaxis defects in the *dnj-14* mutants were too severe to be attributed to the minor defect in locomotion. Observation during the assay revealed that the *dnj-14* mutant worms appeared to move normally across the agar plates, albeit in a random direction, suggesting they were unaware of the presence of the food source. Moreover, quantification of locomotion via thrashing assays revealed no significant defect until later in the worm’s lifespan, before which time the chemotaxis defects are already prominent. Furthermore, chemotaxis defects persist in the heterozygous *dnj-14* mutants, in the absence of any detectable defects in locomotion. We therefore conclude that mutation of *dnj-14* preferentially impacts on chemosensory neurons, with relatively small age-dependent effects on motor function. Given that we could find no functional defects in mechanosensory neurons or in the pharyngeal nervous system across different ages, this suggests that the neuroprotective function of CSP in *C. elegans* is especially important for chemosensory neurons. In this regard, it is noteworthy that selective impairment of distinct neural pathways is also a feature of ANCL, which lacks any observable effects on the visual system, in contrast to the blindness associated with most other NCLs. Similarly, CSPα KO mice exhibit selective degeneration of GABAergic neurons in the hippocampus, with no effect on neighbouring glutamatergic neurons (44). Hence, although CSP has an evolutionarily conserved neuroprotective role, the particular class of neurons that are most susceptible to impaired CSP function varies in different organisms.

We identified a large lifespan reduction in heterozygous *dnj-14(null)/+* worms, in addition to a small defect in chemotaxis, indicating a degree of haploinsufficiency of CSP. This differs from findings from heterozygous CSP^+/−^ mice, which were reported to not impair survival (29). However, this lifespan analysis was only carried out until post-natal day 80, which is around 10% of the maximum lifespan of wild-type laboratory mice. Therefore, it is possible the experiment was not carried out for long enough to identify a reduction in longevity. Furthermore, CSP^+/−^ mice display a loss of spontaneous locomotor activity and thus are not an exact phenocopy of wild-type, indicating that CSP haploinsufficiency impacts on at least one phenotype in mammals (29). Notably, heterozygous *dnj-14(null)/+* worms exhibited a relatively mild chemotaxis defect, whereas they exhibited a large reduction in the lifespan when compared with the ANCL mutant *dnj-14* worms. This indicates that in terms of lifespan, the loss of one copy of CSP has greater consequences than ANCL-causing mutations. This, in turn, suggests that L115R and L116Δ are not complete loss-of-function alleles, consistent with studies of the analogous mutations in a *Drosophila* ANCL model (35). Interestingly, ANCL-causing mutations have been recently reported to have no effect on CSP’s role in endolysosomal microautophagy (19), which could potentially explain the differential effects of heterozygous null and ANCL mutants. Indeed, autophagy has long been established as a major modulator of longevity in *C. elegans* and other model organisms (45). Alternatively, it is possible that chemotaxis more directly reflects neuronal health, whereas lifespan is likely to be impacted by a multitude of factors and different tissues. Although mammalian CSP is enriched in neurons, it is expressed at high levels in many non-neuronal secretory cell types and at lower levels in almost all cells (9); thus, CSP could potentially have important functions in non-neuronal tissues that can affect lifespan. This latter theory could explain why only a partial rescue of the short lifespan of homozygous *csp*^−/−^*Drosophila* mutants is observed when driving expression of either fly or human CSP with neuronal-specific promoters (35), as this would not restore CSP expression to physiologically relevant non-neuronal cells.

One caveat of our study is that we could not investigate the effects of ANCL mutations on palmitoylation or oligomerization/aggregation of the mutant DNJ-14 protein, as our attempts to raise antisera to DNJ-14 for this purpose were unsuccessful. The role of palmitoylation in human ANCL is controversial, with some studies suggesting that ANCL-mutation-induced reduction in CSPα palmitoylation directly leads to its iron-dependent oligomerization (22), whereas others have reported that palmitoylation of ANCL-mutant CSPα not only occurs but is actually required for oligomerization (23,24). Hence, it would be interesting to know the effect of ANCL mutations on the palmitoylation of worm DNJ-14. Despite the controversy over the role of palmitoylation, it is generally agreed that ANCL mutations lead to oligomerization/aggregation of CSPα. Indeed, analogous mutations in human patient-derived cells, mammalian cell culture models and fly models have been shown to cause oligomerization/aggregation of both human and fly CSP (4,21–23,35,42,43). Although the lack of an antibody to DNJ-14 prevented us from demonstrating oligomerization of the mutant worm protein, our finding that ANCL mutant worms cannot be fully rescued by transgenic human CSP (in contrast to *dnj-14* null mutants) strongly suggests that the mutant DNJ-14 protein interacts with human CSP and impairs its function in a dominant-negative manner, consistent with the co-oligomerization model of the human molecular pathology proposed by Naseri *et al* (22). However, significant future work using new DNJ-14 antibodies or epitope tagging approaches would be required to confirm that ANCL mutations cause worm DNJ-14 to oligomerize/aggregate, as they do to CSP in insect and mammalian systems.

Our focused drug screen identified the anti-epileptic drug, ethosuximide as a potential therapeutic for ANCL. This finding is substantiated by the previously reported improvement of lifespan and chemotaxis phenotypes by ethosuximide in *dnj-14(ok237)* and *dnj-14(tm3223)* null mutant worms (27). Rolipram, which has also been shown to improve chemotaxis in *dnj-14(ok237)* and *dnj-14(tm3223)* mutants (26), had no significant effect on ANCL mutant worms. As *ok237* and *tm3223* are putative null alleles, it is possible rolipram can compensate for loss of *dnj-14*, but is unable to ameliorate the negative consequences of dominant negative ANCL-causing mutations. The precise mechanism by which ethosuximide rescues the ANCL mutant phenotype is unknown. However, it is unlikely to be because of a specific effect on palmitoylation or oligomerization/aggregation of the mutant protein, as ethosuximide also rescues the phenotypes of *dnj-14* null mutants, which entirely lack DNJ-14 protein (27). Hence, it seems most probable that the rescuing effect of the drug comes from the activation of a distinct pathway(s) that acts in parallel to CSP’s neuroprotective mechanism(s). Based on previous findings, this pathway(s) is downstream of the conserved pro-longevity and neuroprotective transcription factor DAF-16/FOXO (27). We were unable to reproduce findings of iron chelators partially rescuing ANCL pathology in cultured neurons (22), through testing their ability to rescue chemotaxis defects in *dnj-14* ANCL mutant worms. However, it is important to stress that the lack of effect seen in this study should not be interpreted as evidence against the proposed therapeutic use of iron chelators for ANCL patients. There are many potential reasons why we may have missed such a rescuing effect. For example, only one concentration was tested in this study, so it may be that higher/lower doses are required. There is also uncertainty about how the worm cuticle affects absorption of the iron chelators, and thus their subsequent bioavailability, given the well-documented issues with drug penetration in *C. elegans* (46). Hence, significant future work is required to properly characterize the effects of iron chelators in the worm model. Nevertheless, the identification of ethosuximide from a small-scale pilot drug screen serves as evidence that the *dnj-14* mutant *C. elegans* generated in this study can potentially be utilized for larger-scale drug screening to identify novel candidate therapeutics for ANCL.

## Materials and Methods

### Nematode culture

*Caenorhabditis elegans* were grown and cultured at 20°C on nematode growth media (NGM, 2% (w/v) agar, 0.3% (w/v) NaCl, 0.25% (w/v) peptone, 1 mM CaCl_2_, 1 mM MgSO_4,_ 25 mM KH_2_PO_4,_ 5 μg mL^−1^ cholesterol). *Escherichia coli* OP50 supplemented with 50 μg/mL kanamycin was used as a food source. Bristol N2 *C. elegans* were used as a wild-type reference strain.

### Generation of homozygous *dnj-14 C. elegans* mutants

Homozygous *dnj-14* mutants were created by CRISPR/Cas9 genome editing. The *dnj-14(null)* strain was generated utilising two single guide RNA (sgRNA) sites at the 5′ (5′-ctgcagaatcttctcatcct-3′) and 3′ (5′-ttgttttaaataaattcctc-3′) ends of *dnj-14* in order to produce a clean deletion of the open reading frame. The *dnj-14(L115R)* and *dnj-14(L116Δ)* alleles were generated using one sgRNA (5′-gcgttcggacttctcacggg-3′), and single stranded oligonucleotides (ssoligos) to repair the template. (*dnj-14(L115R)*-TTGAAGCCTTGGTTCAATGGACATTTTTCGCCTTGGGCCGTCTGACgGG**TGG**CTTCTTCTGCTGCTGTTGTGGTTGTATG; *dnj-14(L116Δ)*—TTGAAGCCTTGGTTCAAATGGACATTTTTCGCCTTGGGCCTAACgGGTGGCTTCTTCTGCTGCTGTTGTGGTTGTATG). This had the effect of introducing either a leucine to arginine substitution at amino acid position 138 (termed L115R, adopting the human nomenclature) or a leucine deletion at amino acid position 139 (L116Δ). Several other bases were mutated (underlined in Figure 1B) in order to prevent the Cas9 from continually cutting the DNA and to introduce an *AgeI* restriction site and remove a *BstUI* restriction site for PCR screening purposes. All of these were silent mutations with the exception of a phenylalanine to leucine mutation at amino acid position 136. This substitution is unlikely to have any effect, as this phenylalanine is very poorly conserved through evolution—indeed, it is the only residue not conserved between human and mouse CSP (in which it is a valine; Figure 1A), and is an isoleucine in other *Caenorhabditis* species. Gonadal injections of recombinant purified Cas9, sgRNA and ssoligos, where applicable, were performed by Magnitude Biosciences (Durham, UK). Successful gene editing was confirmed initially by PCR and restriction fragment analysis, followed by DNA sequencing (see below).

### Generation of heterozygous *dnj-14 C. elegans* mutants

Heterozygous *dnj-14* mutants were produced by initially crossing homozygous FX30236 hermaphrodites carrying the tmC30 balancer (*lon-2*; *mec-10*; *Pmyo-2*::*mCherry X*) onto N2 males to generate balanced heterozygous hermaphrodites. Self-fertilization of this resultant strain produced homozygous *lon-2*; mec-10; *Pmyo-2*::*mCherry X* hermaphrodites. These were again crossed back onto N2 males, generating cross progeny males hemizygous for *lon-2*; *mec-10*; *Pmyo-2*::*mCherry X* (male *C. elegans* carry a single copy of the X chromosome). These males were then further crossed onto *dnj-14* mutant or N2 control hermaphrodites, yielding heterozygous N2/*dnj-14* mutant; *lon-2*; *mec-10*; *Pmyo-2*::*mCherry X* worms. These resultant strains were then allowed to self-fertilize, and heterozygous *dnj-14* mutant, *lon-2*; *mec-10*; *Pmyo-2*::*mCherry C. elegans* were maintained by selecting for non-long worms with red pharyngeal fluorescence. Heterozygosity of all *dnj-14* mutant strains was confirmed by PCR and *AgeI* restriction digests (see below).

### Generation of human CSPα rescue *C. elegans* strains

A synthetic DNA construct of the human *DNAJC5* coding sequence, codon-optimized for expression in *C. elegans*, was created by GeneArt (ThermoFisher, UK). The putative *dnj-14* promoter was made by PCR cloning into the PD117.01 vector with the GFP coding sequence removed, as previously described (26). The synthetic *DNAJC5* construct was then sub-cloned into this vector, creating a plasmid with human CSPα expressed under control of the natural worm *dnj-14* promoter. The plasmid was then microinjected into the gonads of N2 hermaphrodites, along with a P*rab-3*::GFP reporter to identify transgenic progeny. GFP-positive hermaphrodite progeny carrying the P*dnj*-14::hCSP; P*rab-3*::GFP extrachromosomal array were then crossed onto N2 males. The resulting cross progeny males were then further crossed onto hermaphrodites homozygous for *dnj-14* mutations and selecting for male offspring with GFP fluorescence. This yielded males hemizygous for the *dnj-14* mutations that also carry the extrachromosomal array P*dnj-14*::hCSP; P*rab-3*::GFP. Backcrossing these males onto hermaphrodites homozygous for *dnj-14* mutations and selecting for GFP fluorescence in the progeny generated hermaphrodites homozygous for *dnj-14* mutations, carrying the extrachromosomal array P*dnj-14*::hCSP; P*rab-3*::GFP.

### Genotype validation of *dnj-14* mutant *C. elegans*

Inheritance of the *dnj*-14 null allele was verified through PCR primers (Forward: 5’-GAAAATTGGTGATGATGCTGCAGG-3′; Reverse: 5′- GTCTCCCAGAGGCAGTCGAACAAC-3′), which produces differentially sized products for WT and *dnj-14* null alleles (WT: 2590 bp; *dnj-14(null)*: 1380 bp). Inheritance of the L115R or L116Δ mutation was verified through initial amplification of a portion of *dnj-14* containing the point mutation, with PCR primers (Forward: 5′-AATCGAGGCGGGAAGGAAAG-3′; Reverse: 5′-TGGGTGTGTCCATTCCGAAC-3′) with subsequent *AgeI* restriction enzyme digestion, which distinguishes between WT and *dnj-14* mutant alleles, based on the presence/absence of digestion (WT: 1449 bp; *dnj-14(L115R)*/*dnj-14(L116Δ):* 557 + 892 bp). The presence of the desired ANCL mutations was further validated through sequencing (DNA Sequencing and Services, University of Dundee, UK). As CRISPR/Cas9 gene editing does not generate off-target events to any significant degree in *C. elegans* based on whole genome sequencing analysis (47), out-crossing is unnecessary. Hence, homozygous mutant strains were used for phenotypic analysis without further out-crossing; heterozygous worms and strains rescued with human CSPα were out-crossed once with N2 prior to use in phenotypic assays. All strains are freely available upon request. The official names for the new alleles created in this study are as follows: null = *dnj-14(ulv20);* L115R = *dnj-14(ulv21);* L116Δ = *dnj-14(ulv22).*

### Age-synchronization

*Caenorhabditis elegans* were age-synchronized through either bleaching or timed-egg lays. For bleaching, populated plates of adult worms were washed in two parts 8% commercial alkaline hypochlorite bleach and 1 part 5 M NaOH. Eggs were released from gravid adult worms through lysis by vortexing the mixture every 2 min for 10 min, followed by pelleting via centrifugation for 1 min at 400 g. Bleach was diluted by washing the pellet in dH_2_O, centrifuged for 1 min at 400 g, with the egg pellet deposited onto a fresh seeded NGM plate. For timed egg-lays, 10–30 adult *C. elegans* were left on seeded NGM plates, to allow for synchronized egg-laying, after which the adults were removed from the NGM so that only synchronized eggs remained. Day 1 adult *C. elegans* were passaged onto new NGM daily to attain synchronized populations of older adult worms of defined ages.

### Lifespan assay

*Caenorhabditis elegans* were age-matched through either bleaching or timed-egg lay. Between 100 and 200 adult day 1 *C. elegans* were collected per strain, then checked for survival every 1–2 days. *Caenorhabditis elegans* were transferred onto new plates every 1–2 days during the first 14 days of the assay, to prevent confusion between the age-synchronized *C. elegans* and their progeny, after which time reproduction ceases. Following this, *C. elegans* were transferred onto new plates prior to OP50 depletion. Escaper or bagged *C. elegans* were censored from the study. Lifespan results were pooled from three independent biological repeats, with a minimum *n* number of 47 uncensored *C. elegans* accounted for at the end of each lifespan repeat.

### Chemotaxis assay

Worms were washed twice, through placing in M9 solution (22 mM KH_2_PO_4_, 42 mM Na_2_ HPO_4_, 85.5 mM NaCl, 1 mM MgSO_4_) with 0.1% BSA (Bovine Serum Albumin) and allowing to thrash for 30 min. This pre-incubation step was included to help wash away OP50 bacteria from the worms, in order to improve signal-to-noise ratio in the assay. Washed *C. elegans* were placed 30 mm away from a 30 μL droplet of OP50, seeded 48 h previously. The number of *C. elegans* reaching the food was recorded every 10 min for 120 min. For age-dependent food race assays, *C. elegans* were age-synchronized and assayed at various stages of adulthood. A minimum of 30 worms per strain were assayed for each timepoint tested, from at least three independent biological repeats.

### Locomotion

Locomotion was quantified through thrashing, where a single thrash was quantified as one complete sinusoidal movement from maximum to minimum amplitude, and back again. A single worm was placed in 50 μL M9 solution with 0.1% BSA and allowed to acclimatize for 10 min prior to recording the number of thrashes per minute. *Caenorhabditis elegans* were age-synchronized and assayed at various stages of adulthood. Thirty worms per strain were assayed for each timepoint tested, from three independent biological repeats.

### Drug screening

All chemicals were obtained from Sigma-Aldrich (St Louis, MO). Compounds were dissolved in dimethyl sulfoxide (DMSO), then added to molten NGM prior to pouring into petri dishes. NGM dishes containing the same quantity of DMSO (0.1%) were used as a vehicle control. Where drug vehicles were not required (ethosuximide was dissolved directly in NGM), DMSO was added separately to 0.1% regardless, to ensure consistency between conditions tested. Drug concentrations used are described in Supplementary Material, Table S1. Worms were maintained on chemical plates from the point of egg-lay up until the worms were assayed (between 1 and 4 days of adulthood) by chemotaxis assays described above. A minimum of 30 worms per strain were assayed per drug condition, from a minimum of three independent biological repeats.

### Effect of ethanol on locomotion

Locomotion was quantified through thrashing, where a single thrash was defined as one complete sinusoidal movement from maximum to minimum amplitude, and back again. A single *C. elegans* worm was placed in 0 or 17 mM ethanol in 200 μL M9 solution with 0.1 mg/mL BSA, in a 35 mm petri dish with the lid on and secured with parafilm to reduce ethanol evaporation. The worms were allowed to acclimatize for 10 min, thereafter the number of thrashes per minute was recorded. *Caenorhabditis elegans* were assayed at day 2 and 5 of adulthood. Fourty worms per strain were assayed for each ethanol condition and timepoint tested, from three independent experimental repeats.

### Electropharyngeograms

EPGs, used to analyse pharyngeal neural and muscular activity were acquired with the NemaMetrix ScreenChip system (Nemametrix Inc, Eugene, Oregon), as described previously (38). Pharyngeal electrical activity was recorded for a minimum of 180 s, with relevant parameters being monitored, including the mean pump frequency (Hz) and interpump interval (IPI) duration. EPG data were acquired and analysed using NemAcquire and NemAnalysis software. For age-dependent pharyngeal pumping assays, *C. elegans* were assayed at day 2 and 5 of adulthood. 30 worms per strain were assayed at each timepoint tested, from three experimental repeats.

### Seizure assay

This was performed as described previously (37). Briefly, worms were incubated with 7 mg/mL pentylenetetrazol (PTZ) in M9 buffer containing 0.1% BSA for 20 min. Following incubation, the number of head-bobbing convulsions occurring in 30 s were recorded. Worms were assayed at day 2, 6 and 10 of adulthood. Thirty worms per strain were assayed from each timepoint tested, from three independent experimental repeats.

### Mechanosensation

Mechanosensation was assayed through stimulating anterior touch response by gently stroking just posterior to the pharynx, with an eyebrow hair sterilized in 70% ethanol. A reversal was classified as a rapid sigmoidal movement, of at least two cycles, away from the stimulus. Whereas non-reversal was defined either as a slow uncoordinated movement away from the stimulus, or no response. *Caenorhabditis elegans* were assayed at day 2, 6 and 10 of adulthood, with 30 worms per strain assayed at each timepoint tested, from three independent experimental repeats.

### Self-brood size assay

Age-synchronized adult day 1 adult *C. elegans* were placed on individual 35 MM seeded NGM plates, after washing in M9 buffer to remove residual eggs or larvae. The individual adult worms were transferred onto new NGM plates daily, until no progeny was produced for two consecutive days. Post-48 h removing the worms from the plates, the number of offspring were counted and totalled, as an indicator of the total self-brood size. A minimum of eight independent experimental repeats per strain were carried out.

### Statistical analysis

Statistical analysis of *C. elegans* behavioural assay results was generally performed using a one-way analysis of variance with post-hoc tests employing Tukey correction for multiple comparisons. Chemotaxis assays were analysed by long-rank test using GraphPad Prism; and lifespan assays were analysed using the Online Application for the Survival Analysis (https://sbi.postech.ac.kr/oasis/introduction/) (48). Statistical analysis of EPGs, developmental assays and self-brood size assays utilized unpaired *t*-tests. An error probability level of *P* < 0.05 was accepted as statistically significant; however, exact *P*-values for each statistical test are indicated in each figure and legend.

## Supplementary Material

Supplementary_data_ddac263
