# Supplementary material for: A Caenorhabditis elegans model of autosomal dominant adult-onset neuronal ceroid lipofuscinosis identifies ethosuximide as a potential therapeutic
Source: Hum Mol Genet. 2022 Oct 25;32(11):1772–85. doi: 10.1093/hmg/ddac263 (PMC10196665; doi:10.1093/hmg/ddac263)
Supplement: Supplementary_data_ddac263 [file supplementary_data_ddac263.pdf]

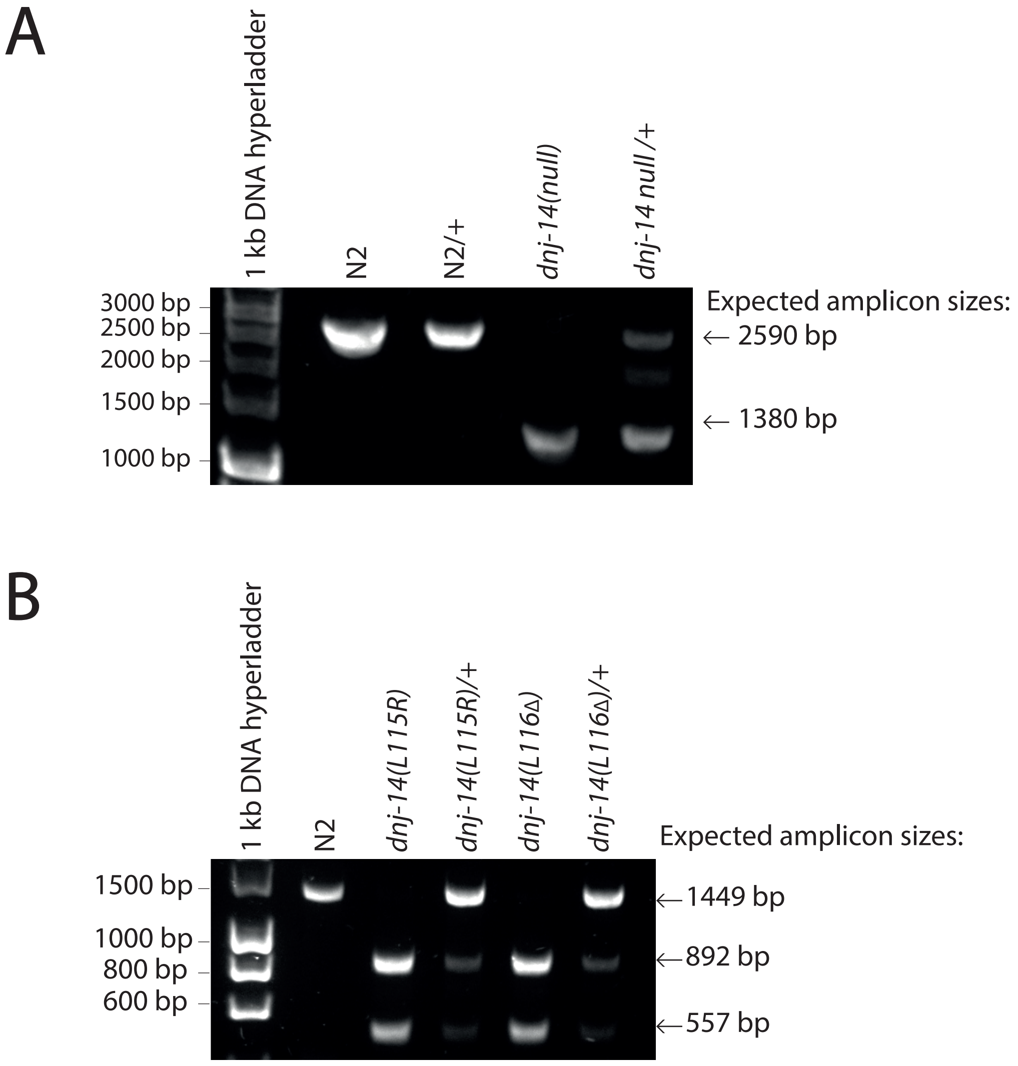

Supplementary Figure 1. PCR Genotyping of *C. elegans* used in this study.
(A) Confirmation of deletion of *dnj-14* in *dnj-14(null)* and *dnj-14(null)/+ C. elegans*, using primers flanking the deletion. (B) Confirmation of successful integration of CRISPR repair template in homozygous and heterozygous ANCL mutant worms, indicated by successful *AgeI* digest. Template was amplified using primers against *dnj-14*, and then subject to an *AgeI* restriction digest.

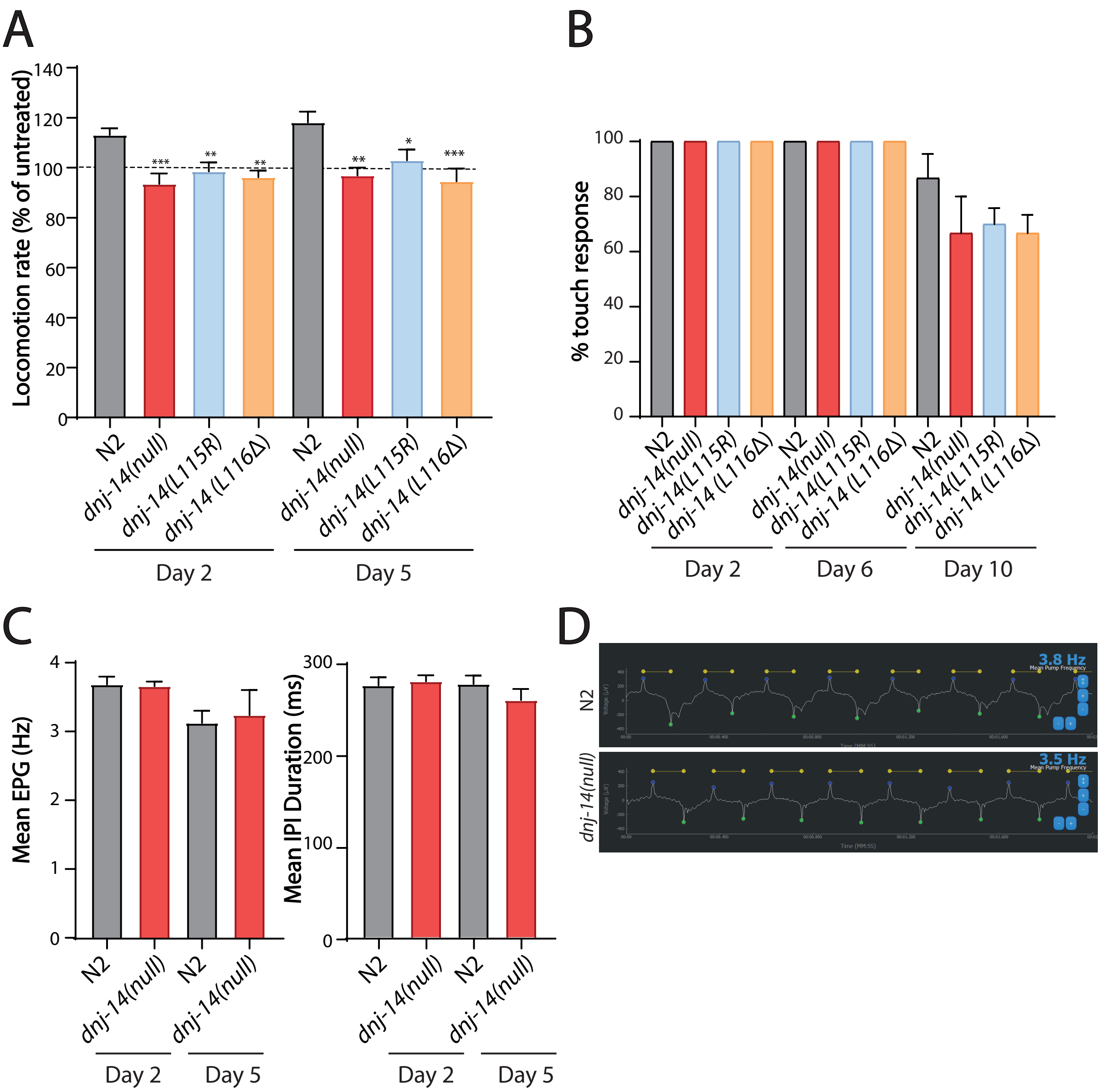

Supplementary Figure 2. Additional assays performed on homozygous *dnj-14* mutants. (A) *dnj-14(null)*, *dnj-14(L115R)* and *dnj-14(L116Δ)* worms have significantly lower rates of locomotion at 17 mM ethanol, compared to N2 wild-type control worms (****P*<0.001, ***P*<0.01 and ***P*<0.01 respectively at adult day 2). Locomotion was determined by thrashing in solution per minute. Data are expressed as normalised to 0 mM ethanol controls. Data are shown as mean ± SEM, with 40 *C. elegans* assayed per strain per timepoint from 4 independent biological repeats. (B) *dnj-14* mutant worms do not exhibit defects in mechanosensation, determined by anterior touch response and measured as percentage of positive responses, indicated by a rapid sinusoidal movement backwards. 30 *C. elegans* were assayed per strain per timepoint, from 3 independent biological repeats. Data are shown as mean ± SEM. (C) *dnj-14(null)* worms do not exhibit a reduction in mean pump frequency (Hz) or inter-pump-interval (IPI) duration, compared to wild-type N2 control worms, at either day 2 or 5 of adulthood. (D) Representative electropharyngeogram (EPG) trace from adult day 2 N2 wild-type control and *dnj-14(null)* *C. elegans*, showing overall EPG recording information such as waveform, mean pump frequency, pump duration, and amplitude. 10 worms were assayed per strain, and data are shown as mean ± SEM.

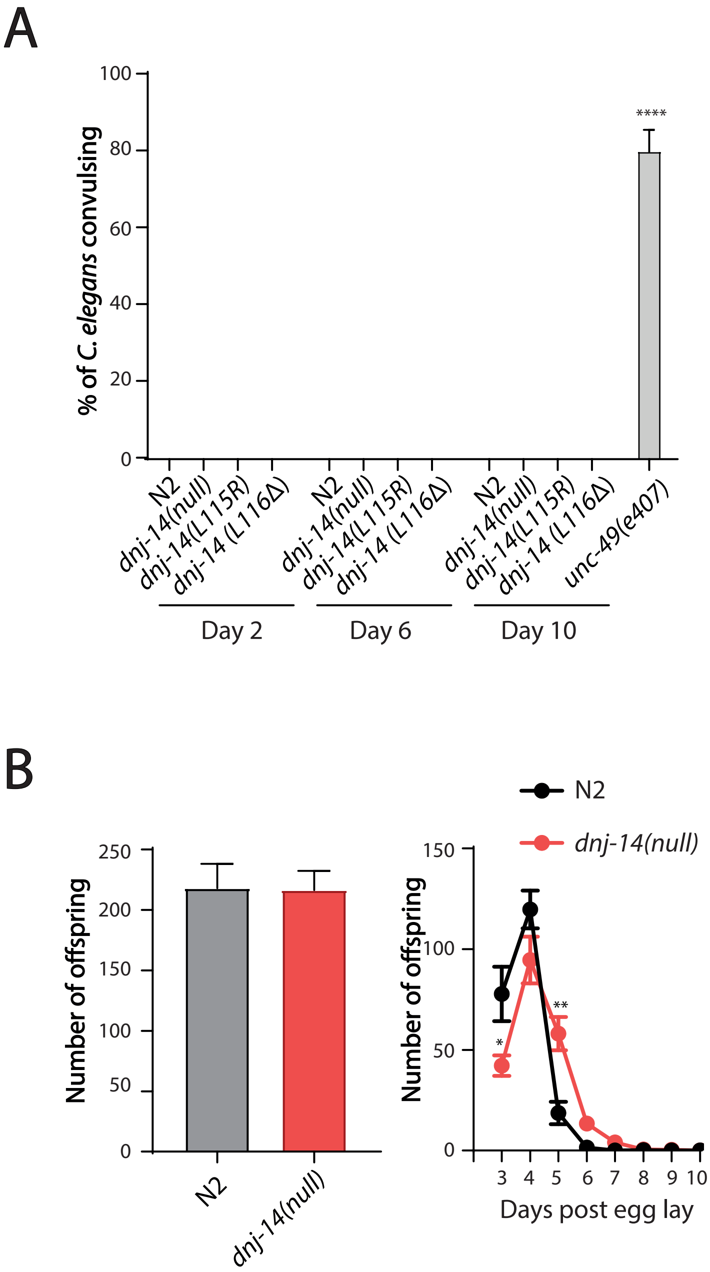

Supplementary Figure 3. Additional assays performed on homozygous *dnj-14* mutants. (A) *dnj-14* mutant worms do not exhibit pentylenetetrazole (PTZ)-induced seizure-like activity. Percent of *C. elegans* convulsing following 20 minute exposure to 7 mg/mL, utilising unc-49(e407) GABA A receptor mutant worms as a positive control. 30 *C. elegans* were assayed per strain per timepoint, from 3 independent biological repeats. Data are shown as mean ± SEM. (B) *dnj-14(null)* worms do not exhibit a reduction in total self-brood size compared to wild-type N2 worms. *dnj-14(null)* worms have significantly smaller self-brood size 3 days post egg lay ($P<0.05$), however, *dnj-14(null)* worms have a significantly larger self-brood size by adult day 5 ($P<0.01$). Represented as both mean total self-brood size and mean brood size per day from 3 days post egg lay. Each assay was carried out with a single *C. elegans*, with a minimum of 8 independent biological repeats. Data are shown as mean ± SEM.

Supplementary Materials and Methods

Supplementary Table 1. List of compounds and corresponding concentrations used.

Compound	Assay	Concentration	Drug Class
Ethosuximide	Drug rescue	1 mg/mL	Anti-epileptic drug
Rolipram	Drug rescue	100 µg/mL	Phosphodiesterase inhibitor
Deferiprone	Drug rescue	28 µg/mL	Iron chelator
Deferoxamine	Drug rescue	112 µg/mL	Iron chelator
Pentylenetetrazole	Seizure assay	7 mg/mL	Non-competitive GABA antagonist
Serotonin	Electropharyngeograms	1.76 mg/mL	Neurotransmitter / hormone
